# Comparison of pulmonary vein reconnection patterns after multielectrode phased radiofrequency- and cryoballoon ablation of atrial fibrillation

**DOI:** 10.1186/s12872-020-01459-4

**Published:** 2020-04-23

**Authors:** Marcus Wieczorek, Kiarash Sassani, Reinhard Hoeltgen

**Affiliations:** 1 School of Medicine; Department of Cardiology and Electrophysiology, Witten/Herdecke University, St. Agnes-Hospital Bocholt, Barloer Weg 125, 46397 Bocholt, Germany; 2 St. Agnes-Hospital Bocholt, Barloer Weg 125, 46397 Bocholt, Germany

**Keywords:** Atrial fibrillation, Pulmonary vein isolation, Phased radiofrequency, Cryoballoon, Pulmonary vein reconnection

## Abstract

**Background:**

Pulmonary vein isolation (PVI) using phased radiofrequency (RF) energy has been shown to be effective in the treatment of atrial fibrillation (AF).

**Methods:**

We characterize and compare pulmonary vein (PV) reconnection at repeat ablation in patients with AF after initially successful PVI using phased RF technology (PVAC) or 2nd generation cryoballoon (CB). Eighty five patients undergoing redo PVI using multielectrode PVAC phased RF catheter and 66 patients after CB PVI were enrolled 9.7 ± 3.4 months after the initial ablation procedure.

**Results:**

The percentage of patients with PV reconnection(s) was comparably high between both groups (93% PVAC and 92% CB). However, 75% of all PVs and left common trunks (CTs) isolated with PVAC were reconnected, compared with 52% reconnections after CB PVI (*p* < 0.001). A mean of 2.79 ± 1.2 PVs and CTs/patient were reconnected after PVAC PVI compared with 1.97 ± 0.8 in CB patients, *p* < 0.0001. No patients in the CB group had 4 reconnected PVs, while this pattern of reconnection was observed in 33% in the PVAC group (*p* < 0.0001). The percentage of patients in the PVAC group with ≥3 reconnected PVs was significantly higher compared with CB patients (56 patients (66%) vs. 17 patients (26%), *p* < 0.0001), while the percentage of patients with no PV reconnection was comparably low in PVAC and CB patients (7 and 8%, respectively). CTs were most frequently reconnected after PVAC PVI (94%) and left superior PVs after CB ablation (67%), respectively.

**Conclusions:**

The number of patients with recurrent AF and PV reconnection(s) at redo PVI was comparably high between both groups. However, the extent and distribution of PV reconnections was different in many aspects, indicating more stable atrial lesions after CB PVI compared with PVAC technology.

## Background

There is general consensus about the efficacy of pulmonary vein isolation (PVI) in the treatment of atrial fibrillation (AF) with catheter ablation procedures. Recovery of pulmonary vein (PV) to left atrium (LA) conduction is the typical electrophysiological finding in patients with recurrence of AF after an initial ablation procedure [1, 2]. Different PV reconnection rates between 19 and 64% have been reported in the past, depending on the ablation technology and the targeted PVs [3, 4]. In case of proven reconnection, repeat PVI can result in a significant improvement in long-term ablation success [5].

The purpose of this study was to characterize and compare PV reconnection patterns at repeat electrophysiological study in patients with AF recurrences after initially successful PVI with phased multielectrode radiofrequency technology (PVAC) or 2nd generation cryoballoon (CB). Both have shown to be safe and effective in PVI procedures [6, 7]. While phased RF technology aims to achieve PVI by duty-cycled uni/−bipolar RF energy using a multipolar circumferential catheter, cryoballoon PVI is designed as a true single shot device to create circumferential transmural lesions after transient complete PV occlusion.

## Methods

### Patients

From a group of 965 patients with a first PVI performed in our institution between May 2013 and June 2018 all patients undergoing a second electrophysiology study in our institution for AF recurrences after completing a blanking period of 3 months were evaluated retrospectively for this study to determine and compare the patterns of PV-LA reconnection, if present. PVI was initially performed with PVAC in 620 patients and with CB in 345. Only patients with regular follow-up visits in our institution until recurrence of AF were recruited for data analysis. Energy sources for first PVI in our institution were multielectrode phased radiofrequency (PVAC or PVAC Gold, Medtronic, CA, USA) in 85 patients and 2nd generation CB (Arctic Front Advance, Medtronic, MN, USA) in 66 patients. Patients with a need for touch-up ablation during the first procedure (6% for PVAC and 5% for CB) were excluded from this study. The ablation technique used for the index procedure was at the operator’s discretion.

### Index procedure

#### Phased radiofrequency PVI

Our general ablation protocol for PVI using the PVAC has been described in detail elsewhere [8–10]. In order to reduce the higher incidence of clinically silent cerebral microembolism by PVAC, [11] a submerged loading of PVAC into the introducer was routinely practiced since November 2013 and care was taken to avoid electrical interactions between PVAC electrodes 1 and 10, as they contribute to the incidence of cerebral microembolism. PVAC and PVAC GOLD are 9-F, over-the-wire, steerable circular mapping and ablation catheters used in combination with a multichannel RF generator (GENius, Medtronic, CA, USA). The system can synchronously apply duty-cycled phased unipolar/bipolar RF energy over 10- (PVAC) or 9 (PVAC GOLD) electrodes, where bipolar refers to adjacent electrodes. After written informed consent, PVAC was inserted into the LA using a long steerable 9Fr sheath (Greatbatch MedicalTM, MN, USA) and directed over a guidewire placed in each targeted PV towards the PV antrum. We recorded bipolar electrograms of PV potentials over all electrodes. Under deep analgosedation, ablation was always started with a 2:1 bipolar/unipolar ablation mode. It was at the operator’s decision to convert to a 1:1 mode, if PVI could not be achieved. Several overlapping RF applications, with a maximum duration of 60 s each, were made while repositioning the PVAC after each application, until all PV potentials were eliminated. PVAC was also used for validation of bidirectional PV conduction block after a waiting period of 30 min.

#### Cryoballoon PVI

After written informed consent, CB ablation was performed under mild conscious sedation, as previously described by our group [10]. A 15- or 20-mm-diameter inner lumen catheter (Achieve™, Medtronic, MN, USA) was placed in each targeted PV to record PV signals before, during and after ablation. Through a steerable sheath placed in LA (FlexCath™, Medtronic, MN, USA), the 28-mm or 23 mm CB (Arctic Front Advance™, Medtronic, MN, USA) was advanced over the Achieve catheter, inflated, and then directed to each PV ostium. Optimal vessel occlusion was assumed when contrast injection into the PV showed complete contrast retention without any backflow to the atrium. A single freeze protocol with a standard duration of 240 s at the beginning and later with 180 s duration was used with a target temperature of − 40 °C and/or PV isolation within one min. Additional freezes, with different CB positions were applied if the first freeze was not successful or in the case of nadir temperature greater than − 40 °C. Phrenic nerve stimulation was performed during ablation of the right-sided PVs to minimize the risk of phrenic nerve palsy by a steerable multipolar catheter placed in the superior vena cava (cycle length 1000 ms, 15 mA/3.0 ms pulse width). To distinguish atrial far-field signals from PV potentials, coronary sinus pacing from different sites was performed, if necessary. PVI with verified entrance and exit block or absence of any electrical signal for ≥30 min was performed by the achieve catheter [12].

#### Definition and ablation strategy of left common trunk (CT)

We defined a CT as a bifurcated PV entering the LA contour together at a distance between the virtual border of the LA and the bifurcation of both PVs > 5 mm.

CT isolation with CB: If an antral occlusion of the CT, validated by contrast injection, could be obtained, a freeze was initiated. If an antral CT occlusion could not be obtained, a sequential ablation approach targeting each PV branch was used.

CT isolation with PVAC: to avoid PV stenosis of the targeted CTs, PVAC was always directed to the antral aspect of the CTs. Multiple PVAC rotations around the CT ostium followed by energy applications were generally performed, until CT isolation was achieved.

#### Repeat ablation procedure

All procedures were performed after informed consent and started under conscious sedation. A hexapolar electrode catheter was placed in the coronary sinus (CS) for pacing and bipolar recordings. After transseptal puncture, a long steerable sheath (FlexCath or Greatbatch Medical).

was inserted into the LA to delineate all PVs angiographically. All PVs were then assessed for PV reconnection. PV reconnection was determined at the onset of the procedure using one of the following multipolar circular catheters: 20 mm inner lumen mapping catheter Achieve (Medtronic, Minneapolis, MN, USA), Lasso (Biosense Webster Inc., Diamond Bar, CA, USA) or Advisor (Abbott, St. Jude Medical, St. Paul, MN, USA), as preferred by the operator. The catheters were placed in each PV with evaluation for PV potentials. Electric isolation of each PVs was confirmed by entrance and exit block, assessed by Achieve, Lasso or Advisor catheter positioned at the PV antrum and/or inside the PV, as necessary.

All PVs/CTs with PV reconnection were targeted for re-isolation with PVAC GOLD, CB or open-irrigated RF-energy point-by point, depending on the operator’s preference. Most patients without PV reconnection were offered an individualized antiarrhythmic drug treatment.

### Follow-up strategy

All our patients underwent routine follow-up visits in an outpatient clinic every 3 months after the index ablation procedure or earlier in case of symptoms. In patients with early and/or recurrent symptomatic episodes of AF, antiarrhythmic drug (AAD) therapy was temporarily used and terminated again after completing the blanking period of 3 months or at least 1 week before the first follow-up visit. Amiodarone therapy was terminated immediately after re-ablation. At each follow-up interval, starting 3 months after the initial procedure, a three-day Holter monitor recording was performed without AAD therapy. Patients were asked to contact our hospital in case of ongoing palpitations. We defined freedom from AF as the absence of AF lasting > 30 s.

### Statistical analysis

All continuous variables are reported as mean ± standard deviation (SD) and were compared by student’s t test, Mann Whitney U test or Wilcoxon test. Categorical variables were compared by chi-square or Fisher’s exact method, as appropriate. A two tailed *P* value < 0.05 was considered to be statistically significant. Statistical analysis was performed using GraphPad Prism software, version 8.

## Results

### Study population

A total of 151 patients were included in this study with a mean age of 60 ± 15 years, male dominance (63%), moderate LA enlargement (43 ± 5 mm) and paroxysmal AF in 65%. Lone AF was present in 48% of all patients. Baseline clinical characteristics were similar between both group (Table 1).

**Table 1:** Clinical characteristics of the enrolled patients (*N* = 151)

	Group 1 (PVAC *N* = 85)	Group 2 (Cryo *N* = 66)	*p* value
Age (years)	58 ± 17 [31–72]	63 ± 12 [29–83]	ns
Male	56 (66)	39 (59)	ns
CHA_2_DS_2_-VASc-score	2.2 [0–5]	2.4 [0–5]	ns
Paroxysmal AF	58 (68)	40 (60)	ns
Left ventricular ejection fraction (%)	57 ± 8	54 ± 7	ns
Left atrial diameter (mm)	43 ± 4 [37–50]	42 ± 6 [36–53]	ns
No. of antiarrhythmic drugs	1.4 ± 1.3 [0–3]	1.1 ± 1.2 [0–3]	ns
Amiodarone therapy	17 (20)	12 (18)	ns
No structural heart disease	44 (52)	28 (42)	ns
Hypertension	42 (49)	38 (58)	ns
Coronary artery disease	22 (26)	15 (23)	ns
Chronic obstructive pulmonary disease	9 (11)	7 (11)	ns
Implantable cardiac devices^a^	6 (7)	7 (11)	ns

Data are numbers with mean ± SD, range [] and % in (); ns: no significant difference, ^a^ pacemaker, implantable cardioverter/defibrillator, event recorder

### Follow-up results

Average time until recurrence of AF after ablation was 8.5 ± 3.1 months for all patients. Recurrence time was significantly shorter for PVAC patients compared with CB patients (8.1 ± 3.4 vs. 9.1 ± 2.6, *p* = 0.0066). After a mean of 9.7 ± 3.4 months, a redo ablation procedure was performed in all 151 patients with a shorter time interval until re-ablation for PVAC- (9.2 ± 3.8 months) compared with CB patients (10.4 ± 2.9 months), *p* = 0.0063. However, mean time delay between redetection of AF and re-ablation was not different between both groups (1.1 ± 0.9 months for PVAC and 1.3 ± 0.9 for CB). During follow-up, PVAC patients experienced a mean of 2.3 ± 1.2 AF episodes compared with 1.7 ± 0.8 episodes for CB patients (*p* = 0.0198). The rate of sustained AF episodes with a need for electrical cardioversion was comparable (34% for PVAC, 46% for CB). While 75% of the PVAC patients experienced AF recurrences within 4–9 months after the blanking time, only 56% in the CB group had recurrences within this period (*p* = 0.0151).

### Reconnection patterns at repeat ablation

Electrophysiological characteristics of both groups at the time of re-ablation are presented in Table 2. In both groups the percentage of patients with PV reconnection(s) was comparably high: 93% in the PVAC and 92% in the CB group.

**Table 2:** Electrophysiological characteristics at redo procedure

	Group 1 (PVAC *N* = 85)	Group 2 (CB *N* = 66)	*p* value
Angiographically delineated PVs / CTs	308/17	244/11	–
Targeted PVs / CTs at initial procedure	302/17	242/11	1.0000
Isolated PVs / CTs at initial procedure	298/17	240/10	1.0000
Pts. with PV reconnections	79 (93%)	61 (92%)	1.0000
Reconnected PVs and CTs at redo procedure	237/315 (75%)	130/250 (52%)	< 0.0001
Reconnected left-sided PVs/CTs	125/152 (82%)	75/119 (63%)	0.0005
Reconnected CTs	16/17 (94%)	6/10 (60%)	0.0473
Reconnected LSPV	57/67 (85%)	36/54 (67%)	0.0170
Reconnected LIPV	52/68 (76%)	33/55 (60%)	0.0533
Reconnected right-sided PVs	112/163 (69%)	55/131 (42%)	< 0.0001
Reconnected RSPV	63/79 (80%)	29/65 (45%)	< 0.0001
Reconnected RIPV	49/84 (58%)	26/66 (39%)	0.0321
Reconnected superior PVs	120/146 (82%)	65/119 (55%)	< 0.0001
Reconnected inferior PVs	101/152 (66%)	59/121 (49%)	0.0044
PV reconnections per patient #:	2.79 ± 1.2	1.97 ± 0.8	< 0.0001
Pts. with 4 reconnected PVs	28 (33%)	0 (0%)	< 0.0001
Pts. with 3 reconnected PVs	28 (33%)	17 (26%)	0.2899
Pts. with 2 reconnected PVs	18 (21%)	35 (53%)	0.0001
Pts. with 1 reconnected PV	5 (6%)	9 (14%)	0.2723
Pts. without reconnected PVs	6 (7%)	5 (8%)	1.0000

*PTs* Patients, *CB* 2nd Generation cryoballoon, *PVs* Pulmonary veins, *CTs* Common trunks, *LSPV* Left superior pulmonary vein, *LIPV* Left inferior pulmonary vein, *RSPV* Right superior pulmonary vein and *RIPV* Right inferior pulmonary vein. #: in patients with a reconnected common trunk, CT was considered and counted as one PV

In the PVAC group, 237 out of 315 initially isolated PVs/CTs (75%) were reconnected to LA, while 130 out of 250 PVs/CTs (52%) were reconnected in the Cryo group (*p* < 0.001). As a result, a mean of 2.79 ± 1.2 PVs and CTs/patient were reconnected after PVAC PVI compared with 1.97 ± 0.8 in CB patients, *p* < 0.0001. 94% of the CTs -counted as one PV- in the PVAC group were reconnected, in contrast to 60% in the Cryo group (*p* = 0.0473). Moreover, we found superior- (82 vs 55%), inferior - (66 vs 49%), left-sided - (82 vs 63%) and right-sided PVs (69 vs 42%) more frequently reconnected in the PVAC group, compared with CB patients.

**Fig. 1:**
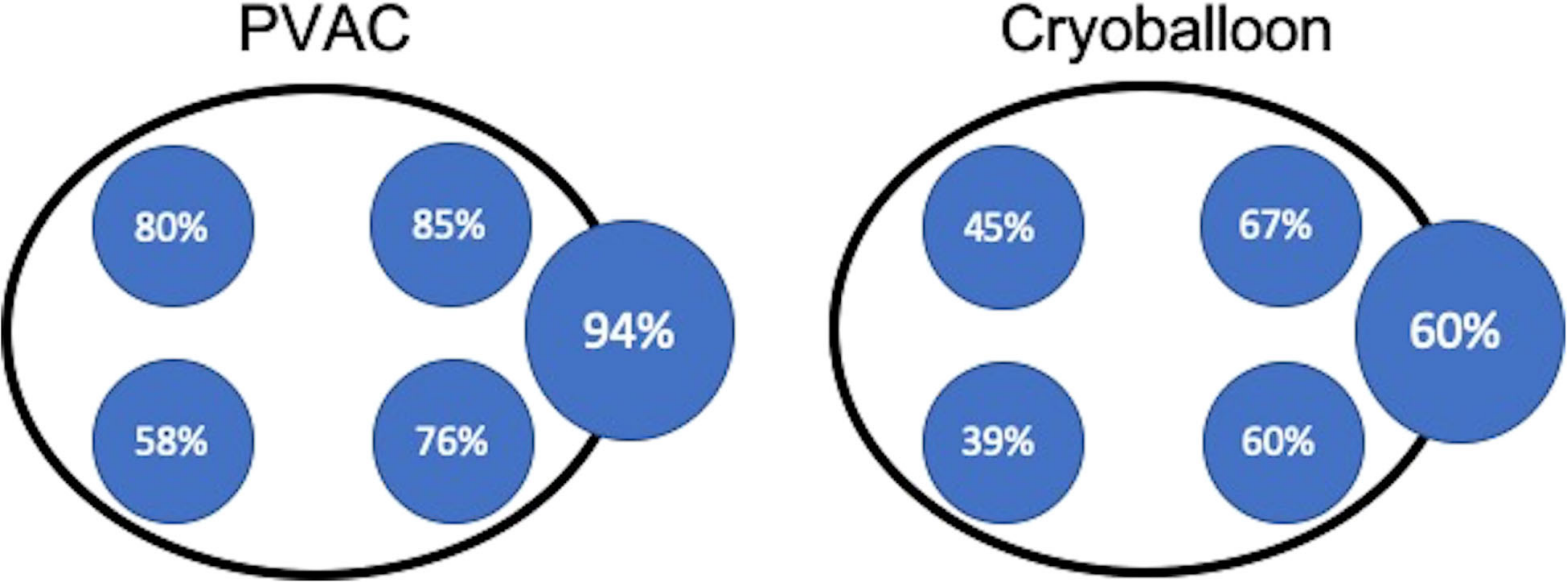
Schematic left anterior oblique view to LA with percentage of reconnection observed for each pulmonary vein / left common trunk after PVAC (left) and Cryoballoon PVI (right)

A detailed analysis of LA-PV reconnections for each PV showed significantly higher reconnection rates in the PVAC group compared with CB patients for left superior pulmonary veins (LSPVs) (85% vs. 67%, *p* = 0.0170), right superior pulmonary veins (RSPV) (79% vs. 45%, *p* < 0.0001) and right inferior pulmonary veins (RIPV) (58% vs. 39%, *p* = 0.0321). In both groups the lowest reconnection rates were observed for the RIPVs (Fig. 1), while the highest rates of reconnection were detected in CTs after PVAC- (94%) and in LSPVs after CB PVI (67%).

The distribution of patients with 0, 1, 2, 3 and 4 reconnected PVs is presented in Table 2 for each group. No patient in the CB group had 4 reconnected PVs, while this pattern of reconnection was observed in 33% of the PVAC group (*p* < 0.0001).

As a result, the percentage of patients in the PVAC group with ≥3 reconnected PVs was significantly higher compared with CB patients (56 patients (66%) vs. 17 patients (26%), *p* < 0.0001), while the percentage of patients with no PV reconnection was comparably low in PVAC and CB patients (7 and 8%, respectively).

If the percentage of *patients* with PV reconnections at redo procedure is calculated for each vein (Table 3), there were more patients with reconnected right-sided PVs after PVAC PVI compared with CB treated patients (74 vs 44% for RSPV and 58 vs 39% for RIPV).

**Table 3:** Percentage of patients with reconnection at redo procedure calculated for each vein

	Group 1 (PVAC *N* = 85)	Group 2 (CB *N* = 66)	*p* value
Reconnected CTs	16 (19%)	6 (9%)	0.1077
Reconnected LSPV	57 (67%)	36 (55%)	0.1313
Reconnected LIPV	52 (61%)	33 (50%)	0.1885
Reconnected RSPV	63 (74%)	29 (44%)	0.0002
Reconnected RIPV	49 (58%)	26 (39%)	0.0330

No. of reconnections and percentage () related to each group, CTs Common trunks, *LSPV* Left superior pulmonary vein, *LIPV* Left inferior pulmonary vein, *RSPV* Right superior pulmonary vein and *RIPV* Right inferior pulmonary vein, *CB* 2nd Generation cryoballoon

Reconnection patterns for each ablation device at repeat PVI are presented in Table 4a. For both devices we found higher reconnection rates for left- sided PVs compared with the right-sided. Further, PVAC patients had higher reconnection rates in superior- compared with inferior veins. These effects were not influenced by the high reconnection rates of CTs counted as left-sided PV (Table 4b).

**Table 4:** Comparison of PVAC and CB reconnection patterns

a)	Reconnected left-sided vs right-sided PVs/CTs	Reconnected superior vs inferior PVs/CTs
PVAC	125/152 (82%) vs 112/163 (69%)	136/163 (83%) vs 101/152 (66%)
p value	**0.0061**	**0.0006**
CB	75/119 (63%) vs 55/131 (42%)	71/129 (55%) vs 59/121 (49%)
p value	**0.0010**	0.3754
b)	**Reconnected left-sided vs right-sided PVs**	**Reconnected superior vs inferior PVs**
PVAC	109/135 (81%) vs 112/163 (69%)	120/146 (82%) vs 101/152 (66%)
p value	**0.0235**	**0.0023**
CB	69/109 (63%) vs 55/131 (42%)	65/119 (55%) vs 59/121 (49%)
p value	**0.0012**	0.3696

### Ablation strategy at repeat ablation

In 79 PVAC patients, PVI re-Isolation was successfully performed with PVAC or PVAC GOLD in 40-, CB in 36- and irrigated RF in 3 patients. 61 CB patients with PV reconnections were effectively treated with CB in 42-, PVAC GOLD in 13- and irrigated RF in 6 patients.

## Discussion

### Main Findings

We analyzed LA-PV reconnection patterns in patients with recurrent AF undergoing repeat PVI. Initial PVI had been performed by duty-cycled phased radiofrequency in 85- (PVAC) and 2nd generation CB in 66 patients. After a mean of 9.7 months, a comparably high percentage of patients (93% PVAC and 92% CB) had PV reconnection in one or more PVs. However, there were far more reconnected PVs and CTs after PVAC PVI compared with CB patients and the number of patients with ≥3 reconnected PVs was significantly higher in PVAC patients. CTs and LSPVs were most frequently reconnected after PVI with PVAC and CB, respectively. PV reconnection patterns of both groups were different in many aspects.

### PV reconnection patterns after PVAC PVI

PV reconnection is the typical finding in patients with recurrent AF after PVI [1]. Different PV reconnection rates in patients with recurrent AF have been reported in the past for different ablation methods. After PVAC PVI, Rademakers et al. reported a mean of 3.2 ± 0.7 reconnected PVs/patient with 46% of the patients showing reconnections of three PVs. There was no patient without PV reconnection [13]. Similar results after PVAC PVI were reported by Balt et al.: 98% of all patients had PV reconnection(s) at repeat ablation with 34% exhibiting reconnections of three PVs [4]. Similarly, we observed PV reconnections in 93% of the patients and in 75% of the previously isolated PVs/CTs after PVAC PVI with a mean of 2.79 ± 1.2 reconnected PVs/CTs per patient. 66% of our patients had reconnections of three PVs. These results indicate that significant PV/CT reconnections must be expected at repeat ablation after PVAC PVI. The highest reconnection rate (94%) after PVAC PVI was observed for CTs in our study. The same effect was described by Balt et al. with a 83% reconnection rate for CTs [4]. We further found that right-sided PVs were less frequently reconnected after PVAC PVI compared with left-sided PVs, while superior PVs were more frequently reconnected than inferior veins, both scenarios not influenced by the presence of CTs. As already described by others, the anatomical ridge between LSPV and left atrial appendage (LAA) is a preferred location for PV reconnection, irrespective of the ablation technique [14, 15]. Reduced contact of the circumferential PVAC at this location in combination with a thicker atrial wall are possible contributors for a higher PV reconnection rate found for the left superior PVs. High reconnection rates found for CTs in our study after PVAC PVI could be explained with the fixed catheter diameter of 25 mm, which can result in inadequate tissue contact in CT diameters beyond 25 mm. To avoid any RF delivery inside the CT, PVAC must be directed antrally to create multiple overlapping positions until CT isolation is achieved.

### PV reconnection patterns after CB PVI

In our study PV reconnections after CB PVI were most frequently observed in LSPVs. This finding is consistent with data presented by Shah et al. and Glowniak et al. [12, 16], while Aryana et al. found CTs most frequently reconnected after CB PVI [3]. The anatomical ridge between LSPV and LAA, a common reconnection site with increased wall thickness, reduces the creation of a complete transmural lesion and thus may result in frequent reconnections of the left superior PV. Further, using conventional point-by-point radiofrequency ablation makes is sometimes challenging to obtain stable catheter positions with adequate tissue contact in this area. For cryoballoon ablation an adequate positioning of the balloon in the ridge area resulting in sufficient temperature fall can be difficult to achieve, as well.

60% reconnected CTs were observed in our series of patients.

This finding shows the reduced ability of a balloon with a maximum diameter of 28 mm to routinely achieve a complete occlusion of CTs in all patients. A balloon position more distally inside a CT for sequential isolation its superior and inferior branches, harbors the risk of PV stenosis and may result in smaller antral lesions. Thus, in some of our patients, the proximal portion of the common ostium could have regained electrical connection. Of note, and in contrast to some previous reports of persistent PVI, [17, 18] the right inferior PV had the highest isolation rates in our series, yet Westra et al. reported 100% of durable lesions in right inferior PVs in a group of 40 patients after an index CB PVI [19]. A possible explanation could be the routine use of the “hockey stick” and “pull-down” techniques during CB ablation, which was also used in our series of patients, particularly helpful in isolating inferior pulmonary veins [20].

### Comparison of PV reconnection patterns (PVAC vs CB)

While the number of patients with PV reconnections was not different between both groups, more PVs and PVs per patient were found reconnected after PVAC PVI compared with CB patients. More PVAC patients had more than 2 PVs reconnected at the time of redo PVI. Except for the LIPV (*p* = 0.0053), we found all other PVs less frequently reconnected after CB PVI. These data suggest, that phased RF energy for PVI creates less stable lesions compared with CB ablation. The reported higher PV reconnection rates after conventional radiofrequency PVI compared with CB ablation, observed by other authors [3, 21] can be explained by the mechanism of cryoablation itself: in case of complete PV occlusion, a very uniform lesion surrounding the PV ostium is generally created. As most energy sources for PVI have shown to create transmural lesions, the quality of the lesion is mainly dependent on the application method.

This is the first study to compare the effects of phased radiofrequency, using a circular multipolar catheter (PVAC), on PV reconnection at redo ablation with 2nd generation CB. Reconnection patterns at redo PVI after PVAC ablation were very similar with those reported after point-by point ablation and differ from CB reconnection patterns in many aspects. Comparable with reported reconnection rates of 67% after conventional point-by point PVI, [16] we found 75% PVs reconnected, with multiple PV reconnections per patient and 66% of the patients (61%, reported by Glowniak et al. [16]) showing ≥3 reconnected PVs. PVAC lesions around the PV ostium are known to be less homogeneous compared with CB lesions created with 2nd generation CB, which contributes to the observed differences in PV reconnection patterns. Our data further suggest that CB induced PVI is more persistent over time compared with PVAC technology, although clinical data correlating PV reconnection patterns with freedom from atrial fibrillation are somewhat conflicting.

In a meta-analysis Nery et al. could show that after catheter ablation of AF persistent PVI was associated with a reduced risk of AF recurrence. But the effect was only moderate, and PV reconnections were commonly observed, affecting 58% of AF-free patients. Other studies of patients with paroxysmal AF even showed a weaker association [22]. Comparable results with 2nd generation CB were published by Miyazaki et al. They found that the incidence and patterns of PV reconnections after 2nd generation CB ablation were comparable between patients with and those without clinical AF recurrences [23]. It is possible that persistent PVI is not mandatory for all patients to avoid AF recurrence. In a randomized study Dixit et al. compared PVI of only arrhythmogenic PVs with empiric isolation of all PVs.

Study end point was freedom or > 90% reduction in AF burden while taking previously ineffective antiarrhythmic medication. They found that 75% of patients randomized to empirical PVI versus 71% in the arrhythmogenic PV group (*p* = 0.70) had met the endpoint [24]. In contrast, a study of Verma et al. could show that in almost all patients with AF recurrence PV reconnection had occurred. Furthermore, in patients who failed PVI there were more PVs with reconnection(s) compared with patients who maintained in sinus rhythm on antiarrhythmic medication [2]. Irrespective of the uncertainties in the mechanisms of AF, PVI is now a widely accepted endpoint for catheter ablation of AF. However, it remains an anatomic and empirical strategy, rather than a patient-tailored, individual intervention.

### Study limitations

Our study is a retrospective, non-randomized and single-center analysis of AF patients undergoing a second procedure following an index PVI with PVAC or CB. As such, unadjusted confounders may have been present and influenced our results. As this study protocol was non-randomized, we cannot exclude factors which may have confounded either treatment groups. Patients with asymptomatic AF recurrence during follow-up may have been undetected and thus not recruited for analysis. As this would have affected both groups in a comparable way, it is unlikely that this effect could have been a significant source of bias between the two groups. There was no control group of patients without AF recurrences after PVI. Therefore, the pathophysiological impact of our findings with the view of AF recurrences resulting from PV reconnection alone remains unclear. As there was no adenosine challenge during PV mapping, concealed reconnection might remain undetected in some cases which could have affected the presented PV reconnection patterns in both groups. Further, the reported PV reconnection rates at redo PVI in our study may have been overestimated, as there exist some data about the inaccuracy of PVAC and Achieve mapping catheter to falsely classify some reconnected PVs as isolated [25, 26].

## Conclusions

At redo ablation most patients with recurrence of AF after PAVC and CB PVI had PV reconnection. However, the extent and distribution of reconnected PVs were significantly different in many aspects. The use of CB was associated with greater durability of PV isolation and less frequent PV reconnections/patient compared to PVAC ablation. Whether these differences in PV reconnections affect the rate of AF recurrences has to be determined by appropriate prospective studies.

## Data Availability

The datasets during and/or analyzed during the current study available from the corresponding author on reasonable request.

## Abbreviations

PVI: Pulmonary vein isolation
RF: Radiofrequency
AF: Atrial fibrillation
PV(s): Pulmonary vein(s)
PVAC: Phased radiofrequency catheter
CB: 2nd generation cryoballoon
CT(s): Common trunk(s)
LA: Left atrium
LAA: Left atrial appendage
LSPV(s): Left superior pulmonary vein(s)
LIPV(s): Left inferior pulmonary vein(s)
RSPV(s): Right superior pulmonary vein(s)
RIPV(s): Right inferior pulmonary vein(s)
